# Symbolic heart rate transition motifs during nocturnal sleep are associated with diabetic complications in type 2 diabetes

**DOI:** 10.1371/journal.pone.0333067

**Published:** 2025-09-24

**Authors:** Namareq Widatalla, Sona Al Younis, Samit Kumar Ghosh, Ahsan Khandoker

**Affiliations:** 1 Biomedical Engineering & Biotechnology, Khalifa University, Abu Dhabi, UAE; 2 Healthcare Engineering Innovation Group (HEIG), Khalifa University, Abu Dhabi, UAE; Portugal Football School, Portuguese Football Federation, PORTUGAL

## Abstract

Dysfunction in the autonomic nervous system function during sleep is common in type 2 diabetes mellitus and is associated with increased risk of diabetic related complications. While heart rate variability is a standard non-invasive measure of autonomic function, the utility of symbolic heart rate transition motifs to characterize sleep-specific autonomic dysfunction in diabetes has not been explored. Here, we analysed 5-min electrocardiogram segments during daytime and sleep from 35 male participants ((51 ± 17) years old) with type 2 diabetes mellitus. Frequency-domain heart rate variability metrics and 27 symbolic motifs based on 3-beat heart rate transitions were computed. Logistic regression models adjusted for age and body mass index were used to test associations with diabetic complications. The high frequency power of heart rate variability was significantly higher during sleep than daytime (*p* = 0.012), reflecting enhanced parasympathetic activity. Several symbolic motifs showed differential prevalence between sleep and day. In particular, motif [1, 1, −1] was significantly more prevalent during sleep and was independently associated with diabetic complications (β = −1.8, p = 0.023). Motif [1,1,-1] along with [−1, 1, 1], showed strong positive correlations with high frequency power during day and sleep [Day: [1,1,-1]: *r* = 0.70 (*p* = 7.0e-06), [−1,1,1]: *r* = 0.78 (*p* = 4.5e-07)] [Sleep: [1,1,-1]: *r* = 0.77 (*p* = 5.7e-07), [−1,1,1]: *r* = 0.80 (*p* = 3.1e-07)], suggesting a link to vagal tone. The study’s findings provide a novel, non-invasive approach to sleep-based autonomic monitoring in type 2 diabetes mellitus by showing that the symbolic heart rate motifs during sleep reflect unique autonomic patterns associated with diabetic complications. These results demonstrate the diagnostic utility of motif-based HR analysis for diabetes and sleep medicine.

## Introduction

Heart rate variability (HRV) is a non-invasive marker that reflects the activity of the sympathetic and parasympathetic branches of the autonomic nervous system (ANS) [1]. HRV has been used in previous studies as an approximate assessor of ANS [1–3]. Circadian rhythm is a feature of healthy autonomic regulation parasympathetic (vagal) activity normally dominates during sleep, whereas sympathetic activity prevails during awake [4,5]. High HRV and diurnal oscillations caused by the sympathetic nervous system activity during fight-or-flight and the parasympathetic (vagal) system during sleep are indicators of a healthy autonomic function [4,5]. Circadian patterns in HRV are usually characterized as an increase in the low-frequency power (LFp) and very-low-frequency power (VLFp) during the day, indicating higher sympathetic modulation. On the other hand, an increase in the high-frequency power (HFp), which is a measure of parasympathetic activity, increases during nocturnal sleep [6]. This modulation of HRV is important for cardiovascular function and has been linked to a better stress management and sleep quality [7].

Alteration of the normal day-night HRV rhythm has been associated to an increased risk of cardiovascular events, autonomic malfunction, and metabolic disorders, especially in diabetic patients [7,8]. Diabetes is known to affect ANS function, often causing cardiovascular autonomic neuropathy (CAN), which appears as decreased parasympathetic tone and reduced HRV [9–11]. These changes are most evident during sleep, when a reduction in blood flow may indicate warning signs of diabetes related problems [12]. Sleep provides a unique physiological state during which parasympathetic activity predominates and external confounding factors, such as physical activity, posture, and emotional stress, are minimized. This autonomic stability enhances the detectability of hidden abnormalities in vagal tone, making sleep an ideal window for identifying early-stage neural-related diabetic complications in individuals with type 2 diabetes mellitus (T2DM).

Symbolic heart rate (HR) dynamics and non-linear measures have become potential methods to capture the complexity of autonomic regulation, in addition to traditional linear HRV metrics [13,14]. Symbolic HR may reveal patterns not detected by conventional approaches, especially during transitions between sleep and waking states, and provide increased sensitivity to small or transient changes in HR dynamics [15]. In our previous study, we used a First-order Markov model on symbolic HRV to investigate the association between ANS and kidney function in a cohort of heart failure patients. Symbolic HRV revealed that patients with low estimated glomerular filtration rate (eGFR) exhibited different symbolic HRV patterns compared to those with normal eGFR [16].

Fewer studies investigated the differences in HRV patterns between day and night in relation to certain diabetic problems, even though earlier research has revealed decreased HRV in diabetes. Moreover, to our knowledge, no studies have reported on the potential of symbolic HR transition motifs as indicators of sleep-related autonomic dysfunction in diabetes. Consequently, the objective of this study is to investigate how frequency-based HRV components and symbolic HR motifs differ during the day and during sleep. Using logistic regression models adjusted for age and BMI, we further investigated the correlation between these variables and the existence of diabetes complications. This method might develop sleep-based monitoring tools for early detection and improve knowledge of the temporal nature of autonomic dysfunction in diabetes.

## Methods

### Data description

The dataset used in this study is publicly available online [17]. The dataset used in this study was collected by Cheng et al. [18] and made publicly available as Version 4 of the dataset on Mendeley Data [17]. We were not involved in the original data collection process. Upon recruiting the participants, they were informed of the experimental protocol. The participants were asked to sign the written informed consent prior to participating in the study. The data collection was approved by the Institutional Review Board of Suzhou Science & Technology Town Hospital (No. IRB2019045). The details of data collection were discussed previously [18]. Briefly, 60 male inpatients (50 ± 16) years old with T2DM were recruited at the Suzhou Science & Technology Town Hospital. During hospitalization, clinical examinations took place to obtain information related to the participants’ health and metabolic function. On admission, fasting blood and urine samples were collected on the following morning for routine glucose, lipid and kidney tests. Information related to diabetic complications was also recorded. The diabetic complications included: diabetic retinopathy and cataract, diabetic peripheral neuropathy, coronary artery disease and cardiac insufficiency, lower extremity atherosclerosis or stenosis and carotid plaque.

In addition to clinical data, both subjective and objective sleep quality assessments were performed. Objective sleep quality was assessed using the Pittsburgh Sleep Quality Index (PSQI) [15,19], while subjective sleep quality was evaluated by analyzing ECG recordings during sleep using a cardiopulmonary coupling analysis algorithm [17].

ECG records were collected from the participants by using a U.S. Food and Drug Administration (FDA) approved ambulatory single-lead Holter electrocardiogram monitor (DynaDx Corporation, Mountain View, CA, United States). The device is able to record 24-h of ECG with a sampling frequency of 250 Hz. The recording started at 10 pm on the second day of hospitalization, lasting for 24-h. ECG records were then saved as MATLAB files and divided into three.m files: all ECG during 24-h, sleep: ECG during sleep; day: ECG during awake [18]. The ECG signal quality was checked, and seventeen ECG records were excluded due to the low quality, presence of atrial fibrillation or arrhythmias, and short recording time [18]. Due to the exclusion of 17 ECG records, only 43 ECG records were downloaded from the online source for analysis [17].

### Electrocardiogram (ECG) and heart rate variability (HRV) analysis

In this study, MATLAB 2023b was used to upload the sleep and day ECG files from 43 participants. Two segments of 5 minutes of ECG were selected per MATLAB file (day and sleep). The selection of the 5-minute segments was based on the ease of detecting R peaks by using a MATLAB code. We followed the same methods that were mentioned in our study for R-peak detection [20].

**Fig 1:**
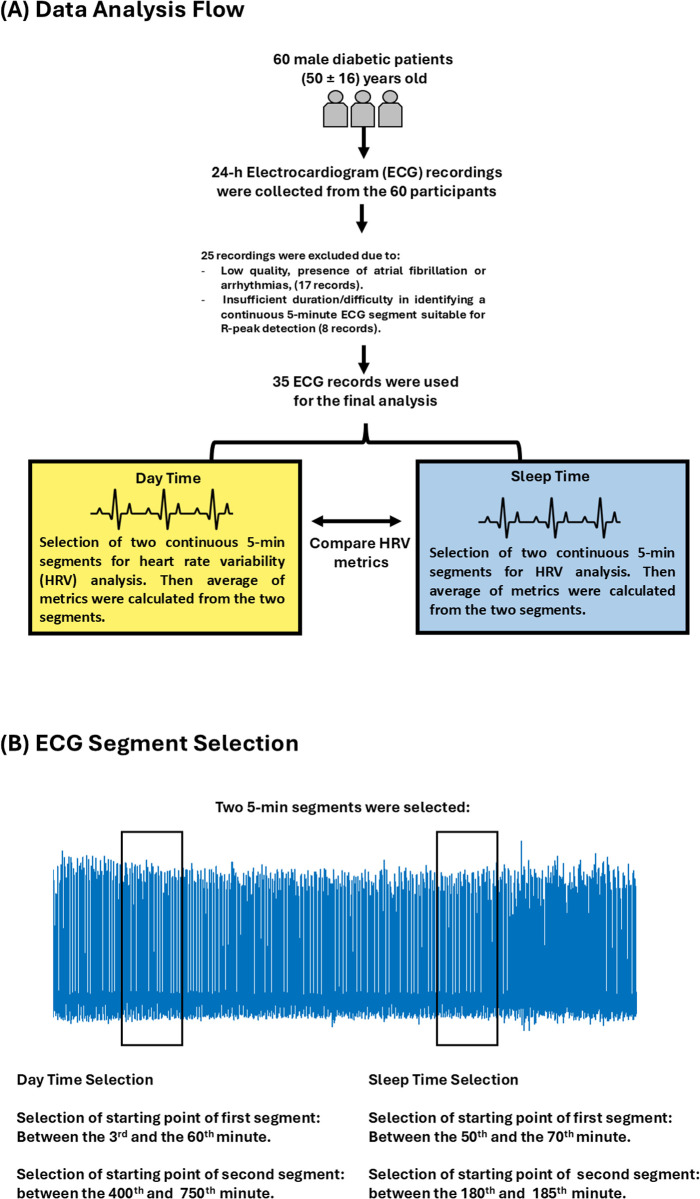
Summary of data analysis. (A) Selection and exclusion criteria of electrocardiogram (ECG) signals are summarized. (B) Selection of ECG segments for daytime and sleep periods is also summarized.

The starting point of each 5-minute segment was randomly selected within predefined time windows. For the daytime (day.m) file, the starting time of the first 5-minute segment was chosen between the 3^rd^ and 60^th^ minute, and the starting time of the second 5-minute segment was chosen between the 400^th^ and 750^th^ minute. For the sleep (sleep.m) file, the starting time of the first 5-minute segment was selected between the 50^th^ and 70^th^ minute, and the starting time of the second 5-minute segment was selected between the 180^th^ and 185^th^ minute. Fig 1B summarizes time selection.

The average of the features calculated from the two segments was calculated. Data from 8 participants were excluded from this study due to the noise that made continuous detection of R peaks for a 5-minute segment challenging. Fig 1 provides a summary of data selection and inclusion. Data from a total of 35 participants were considered for further analysis. Frequency-based HRV analysis was conducted by using the Lomb-Scargle periodogram, considering the following bands [21]: VLFp (0.0033–0.04) Hz, LFp: (0.04–0.15) Hz, HFp: (0.15–0.4) Hz.

- Motif prevalence calculation

A total of 27 Motifs of length 3 were considered in this study by converting RR interval (RRI) into three symbols as follows (the subscript *x* indicates the beat count):

**Symbol −1:** HR deceleration (RR_x+1_ - RR_x_ > 0).
**Symbol 0:** no change in RRI (RR_x+1_ - RR_x_ = 0).
**Symbol 1:** HR acceleration (RR_x+1_ - RR_x_ < 0).

Based on the above definition we calculated the motif frequency of these motifs per subject: [−1,-1,-1], [0,-1,-1], [1,-1,-1], [−1,0,-1], [0,0,-1], [1,0,-1], [−1,1,-1], [0,1,-1], [1,1,-1], [−1,-1,0], [0,-1,0], [1,-1,0], [−1,0,0], [0,0,0], [1,0,0], [−1,1,0], [0,1,0], [1,1,0], [−1,-1,1], [0,-1,1], [1,-1,1], [−1,0,1], [0,0,1], [1,0,1], [−1,1,1], [0,1,1], [1,1,1].

For each motif, the motif prevalence was calculated as follows


$$\text{Motif Prevalance }(\%)=\;\frac{\text{Number of motif occurances}}{\text{Sum of occurances of all other motifs}}\times \text{1}\text{0}\text{0}$$

### Logistic regression

To investigate the association of diabetic complications with HFp and motif prevalence, three models were built to predict the presence of any of the diabetic complications (1) and the absence of complications (0). Model 1 was adjusted for age, BMI and HFp. Model 2 was adjusted for age, BMI, and motif [1, 1, −1] prevalence. Model 3 was adjusted for age, BMI, and motif [−1, 1, 1] prevalence. Only the motifs [1, 1, –1] and [–1, 1, 1] were considered for the logistic regression model because they were the most prevalent compared to the others. Sensitivity analyses were also conducted to assess the robustness of the findings, including models excluding participants with cardiac conditions and those using sleep medication, as well as models adjusting for HbA1c. Results of these analyses are presented in the supplementary material.

### Statistical analysis

Correlation analysis and comparison of means were conducted using Spearman correlation, the Wilcoxon signed rank test [22] and the Friedman test [23] in MATLAB 2023b, respectively. Logistic regression was also performed in MATLAB. In this study, *p-*values less than 0.05 are considered significant.

## Results

### Participant characteristics

Table 1 shows demographic and clinical data of the cohort. The Sleep_quationarie.xlsx file contains PSQI data per subject. In this study, a total of 35 participants were included, with 19 having no diabetes complications and 16 diagnosed with at least one complication. The number of diabetic complications per category was as follows: Diabetic nephropathy: 6, Diabetic retinopathy and cataract: 9, Diabetic peripheral neuropathy: 4, coronary artery disease and cardiac insufficiency: 2, Lower extremity atherosclerosis or stenosis: 2, Carotid plaque: 4.

**Table 1:** Demographic and clinical characteristics of the participants.

Feature	All (*n* = 35)	No Diabetes Complications (*n* = 19)	With Diabetes Complications (*n* = 16)	p – value
**Age (years)**	51 ± 17	46 ± 16	57 ± 16	0.070
**BMI (Kg/m**^**2**^)	27 ± 4.2	28 ± 3.8	25 ± 4.2	0.020
**HbA1c (%)**	8.2 ± 1.6 (*n* = 24)	8.2 ± 1.6 (*n* = 11)	8.2 ± 1.7 (*n* = 13)	0.950
**SBP (mmHg)**	135 ± 15	134 ± 16	136 ± 15	0.730
**DBP (mmHg)**	84 ± 11	85 ± 9.5	82 ± 12	0.280
**WBC (×109/L)**	8.1 ± 4.7	8.8 ± 5.8	7.2 ± 3.1	0.300
**N (%)**	64 ± 13	61 ± 15	66 ± 8.3	0.240
**Hb (g/L)**	147 ± 19	153 ± 18	140 ± 19	0.050
**PLT (×109/L)**	216 ± 57	236 ± 60	192 ± 44	0.020
**CRP (mg/L)**	11 ± 24 (*n* = 30)	11 ± 19 (*n* = 16)	11 ± 29 (*n* = 14)	0.190
**ALT (U/L)**	38 ± 52	50 ± 68	25 ± 15	0.300
**AST (U/L)**	31 ± 40	41 ± 53	20 ± 6.5	0.380
**AST/ALT**	0.88 ± 0.36 (*n* = 34)	0.82 ± 0.32 (*n* = 18)	0.96 ± 0.39 (*n* = 16)	0.280
**GGT (U/L)**	48 ± 49	62 ± 57	32 ± 32	0.009
**BUN (mmol/L)**	5.9 ± 1.9 (*n* = 32)	5.6 ± 2.3	6.2 ± 1.4 (*n* = 13)	0.139
**UA (mmol/L)**	341 ± 99 (*n* = 30)	374 ± 115 (*n* = 17)	297 ± 50 (*n* = 13)	0.039
**TG (mmol/L)**	2.5 ± 2.7 (*n* = 30)	3.6 ± 3.5 (*n* = 15)	1.5 ± 0.72 (*n* = 15)	0.007
**HDL-C (mmol/L)**	1.0 ± 0.25 (*n* = 30)	0.96 ± 0.23 (*n* = 15)	1.1 ± 0.25 (*n* = 15)	0.050
**LDL-C (mmol/L)**	25 ± 0.79 (*n* = 30)	2.8 ± 0.92 (*n* = 15)	2.2 ± 0.50 (*n* = 15)	0.020
**UMA (mg)**	72 ± 97 (*n* = 31)	88 ± 116 (*n* = 15)	56 ± 75 (*n* = 16)	0.330
**UCr (g)**	12 ± 6.3 (*n* = 29)	13 ± 5.8 (*n* = 13)	10 ± 6.5 (*n* = 16)	0.220
**UACR (mg/g)**	7.5 ± 13 (*n* = 29)	5.5 ± 11 (*n* = 13)	9.1 ± 15 (*n* = 16)	0.980

BMI: body mass index, HbA1c (%): glycated hemoglobin, SBP (mmHg): systolic blood pressure, DBP (mmHg), diastolic blood pressure, WBC (× 10^9^/L): white blood cell, N% (%): percent of the number of neutrophils in the number of white blood cells, Hb (g/L): hemoglobin, PLT (× 10^9^/L): platelet, CRP (mg/L): c-reactive protein, ALT (U/L): alanine aminotransferase, AST (U/L): aspartate aminotransferase, AST/ALT: ratio of AST to ALT, GGT (U/L): gamma glutamyltransferase, BUN (mmol/L): blood urea nitrogen, UA (mmol/L): uric acid, TG (mmol/L): triglycerides, HDL-C (mmol/L): high-density lipoprotein-cholesterol, LDL-C (mmol/L): low-density lipoprotein-cholesterol, UMA (mg): urine micro-albumin, UCr (g): urine creatinine, UACR (mg/g): urine albumin-creatinine ratio.

In Table 1, participants with diabetic complications (n = 16) were slightly older than those without complications (57 ± 16 vs. 46 ± 16 years) but this difference did not reach statistical significance (*p* = 0.070). BMI was significantly lower in patients with complications (25 ± 4.2 vs. 28 ± 3.8 kg/m², *p* = 0.020). Hemoglobin A1c values were similar between both groups (8.2 ± 1.7 vs. 8.2 ± 1.6%, *p* = 0.950). Patients with complications had lower hemoglobin levels (140 ± 19 vs. 153 ± 18 g/L, *p* = 0.050) and platelet counts (192 ± 44 vs. 236 ± 60 × 10⁹/L, *p* = 0.020. Gamma-glutamyl transferase levels were significantly reduced in the complication group (32 ± 32 vs. 62 ± 57 U/L, *p* = 0.009). Uric acid was lower in those with complications (297 ± 50 vs. 374 ± 115 mmol/L, *p* = 0.039). Triglycerides were also lower (1.5 ± 0.72 vs. 3.6 ± 3.5 mmol/L, *p* = 0.007), high-density lipoprotein cholesterol was higher (1.1 ± 0.25 vs. 0.96 ± 0.23 mmol/L, *p* = 0.050), and low-density lipoprotein cholesterol – C was lower among those with complications (2.2 ± 0.50 vs. 2.8 ± 0.92 mmol/L, *p* = 0.020). No significant differences were observed in systolic or diastolic blood pressure, C-reactive protein, alanine/aspartate aminotransferase, blood urea nitrogen, urinary albumin, urinary creatinine, or albumin-to-creatinine ratio between groups.

### Frequency-based heart rate variability (HRV) Differences Between Day and Sleep

**Fig 2:**
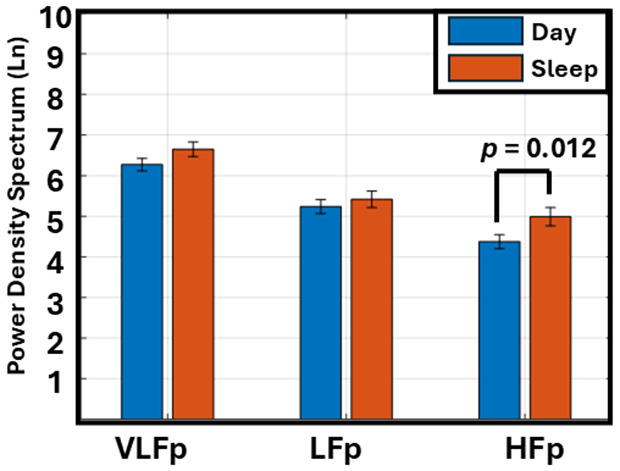
Comparison of frequency-based heart rate variability (HRV) component power between daytime and sleep. Bars represent the mean frequency prevalence of the very low frequency power (VLFp), low frequency power (LFp), and high frequency power (HFp) components during day (blue) and sleep (orange). Error bars indicate the standard error of the mean. There is a significant increase in HFp during sleep compared to daytime (p = 0.012), while no significant differences are noted in LFp (p = 0.860) or VLFp components (p = 0.400).

Fig 2 illustrates the mean prevalence of frequency-based HRV during the day and sleep. A significant (*p* = 0.012) increase in the HFp was observed during sleep (5.0 ± 1.3) compared to daytime (4.4 ± 1.0), reflecting an increase in the parasympathetic activity at night. No significant changes were found in the LFp (*p* = 0.860) or VLFp (*p* = 0.400) between the day and sleep time.

### Symbolic HR transition motif analysis

**Fig 3:**
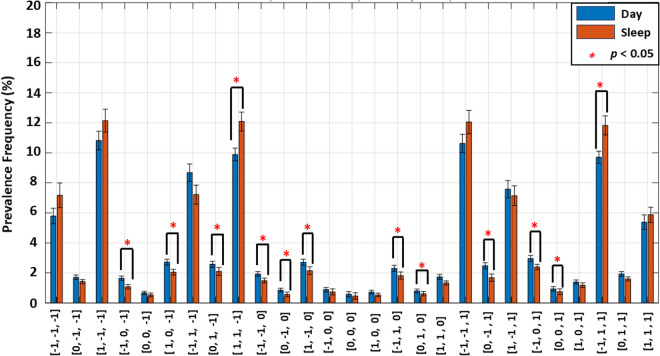
Comparison of motif frequency prevalence (%) between daytime (blue) and sleep (orange) periods. Motifs are 3-beat symbolic sequences derived from heart rate (HR) transitions using values of: HR deceleration −1, no change 0 and HR acceleration +1. Bars represent mean prevalence across subjects, with error bars indicating the standard error (SD) of the mean. Red asterisks (*) denote statistically significant differences between day and sleep periods (p < 0.05). Several motifs, such as [1, 1, −1] and [−1, 1, 1], show significant differences between day and sleep HR transition dynamics.

Fig 3 compares the prevalence of 3-beat symbolic HR transition motifs across day and sleep. Several motifs demonstrated significant changes in prevalence between the two periods. In [−1, 0, −1] (*p* = 3.85e-04), [1, 0, −1] (*p* = 0.028), [0, 1, −1] (*p* = 0.028), [−1, −1, 0] (*p* = 1.3e-03), [0, −1, 0] (*p* = 0.009), [1, −1, 0] (*p* = 0.028), [−1, 1, 0] (*p* = 0.028), [0, 1, 0] (*p* = 0.024), [0, −1, 1] (*p* = 4.1e-03), [−1, 0, 1] (*p* = 0.0013), [0, 0, 1] (*p* = 0.028), the frequency prevalences were significantly higher in the daytime compared to the sleep time. On the other hand, the prevalence of motifs [1, 1, −1] and [−1, 1, 1] were significantly higher in the sleep time compared to the daytime ([1, 1, −1]: *p* = 4.1e-03, [−1, 1, 1]: *p* = 0.012). The differences in frequency prevalence between day and time suggest altered HR transition dynamics between sleep and daytime.

### Associations between HRV features and diabetic complications

Logistic regression models were used to assess the relationship between HRV features and the presence of diabetic complications after adjusting for age and BMI (Table 2). During sleep, the motif [1, 1, −1] was significantly associated with diabetic complications after adjusting for age and BMI (β = − 1.80, *p* = 0.023, 95% confidence interval (95% CI) = [−3.3, −0.17]), whereas no such association was observed during the daytime. Similarly, the motif [−1, 1, 1] showed a trend toward significance during sleep (β = − 1.30, *p* = 0.060, 95% CI = [−2.6, 0.10]), but not during the day. HFp was not significantly associated with diabetic complications during sleep or day. The results in Table 2 highlight that sleep HR transition dynamics are associated with diabetic complications.

**Table 2:** Comparison of daytime and sleep heart rate (HR) transitions and high-frequency (HFp) components in relation to their correlation with diabetic complications.

Feature	Day				Sleep			
	β	*P *– value	95% CI	Model P-value	β	*P *– value	95% CI	Model P-value
**Model 1**								
Age	0.31	0.510	[-0.66, 1.3]	0.10	− 0.30	0.650	[-1.7, 1.1]	0.040
BMI	− 0.70	0.170	[-1.7, 0.34]		−0.85	0.110	[-1.9, 0.23]	
HFp	− 0.21	0.620	[-1.1, 0.67]		− 0.92	0.150	[-2.2, 0.39]	
**Model 2**								
Age	0.45	0.340	[-0.52, 1.4]	0.11	− 0.56	0.380	[-1.9, 0.76]	0.003
BMI	− 0.68	0.180	[-1.7, 0.36]		− 1.10	0.060	[-2.2, 0.09]	
[1, −1]	0.10	0.810	[-0.74, 0.93]		−1.80	0.023	[-3.3, -0.17]	
**Model 3**								
Age	0.34	0.490	[-0.66, 1.3]	0.11	− 0.36	0.550	[-0.97, 0.65]	0.012
BMI	− 0.71	0.160	[-1.7, 0.32]		− 0.95	0.080	[-2.0, 0.15]	
[− 1, 1]	− 0.13	0.760	[-0.98, 0.72]		−1.30	0.060	[-2.6, 0.10]	

BMI: body mass index, HFp: high frequency power, CI: Confidence Interval.

The results of the sensitivity analyses, excluding patients with coronary artery disease and cardiac insufficiency, are reported in S1 Table, and the results after excluding patients taking sleep medication are presented in S2 Table. The logistic regression analysis, including HbA1c as an additional covariate, is shown in S3 Table. In S1 Table, models with HR motifs are significantly associated with diabetes complications during sleep (model with [1,1,-1]: *p* = 0.009, model with [−1,1,1]: *p* = 0.030. S2 Table shows similar results (model with [1,1,-1]: *p* = 0.008, model with [−1,1,1]: *p* = 0.040). In S3 Table, the sleep model with motif [1,1,-1] was the only significant model with *p* = 0.035.

### Correlation between HRV components and motifs

Table 3 shows the correlation between HRV components and the motifs [1, 1, −1] and [−1, 1, 1] across daytime and sleep. Both motifs were strongly positively correlated with the HFp during both day [[1,1,-1]: (*r* = 0.70, *p* = 7.0e-06), [[−1,1,1]: (*r* = 0.78, *p* = 6.3e-04)] and sleep [[1,1,-1]: (*r* = 0.77, *p* = 5.7e-07), [[−1,1,1]: (*r* = 0.80, *p* = 3.1–07)]. LFp was found to be positively correlated with [1,1,-1] and [−1,1,1] during the day only [[1,1,-1]: *r* = 0.51, *p* = 0.002), [−1,1,1]: *r* = 0.56, *p* = 6.3e-04]. The same motifs were not correlated with VLFp, and RRI during sleep or day. The correlation between the motifs and HFp highlights their association with the parasympathetic activity.

**Table 3:** Correlation between HR transition motifs and HRV.

Feature	Day		Sleep	
**[1, −1]**	**[− 1, **1**] **	**[1, −1]**	**[− 1, **1**]**
**VLFp**	0.26 (*p* = 0.130)	0.30 (*p* = 0.310)	0.18 (*p* = 0.290)	0.21 (*p* = 0.220)
**LFp**	0.51 (*p* = 0.002)	0.56 (*p* = 6.3e-04)	0.26 (*p* = 0.330)	0.30 (*p* = 0.080)
**HFp**	0.70 (*p* = 7.0e-06)	0.78 (*p* = 4.5e-07)	0.77 (*p* = 5.7e-07)	0.80 (*p* = 3.1e-07)
**RRI**	0.27 (*p* = 0.120)	0.22 (*p* = 0.200)	0.12 (*p* = 0.480)	0.14 (*p* = 0.400)

VLFp: very low frequency power, LFp: low frequency power, HFp: high frequency power, RRI: RR interval.

## Discussion

This study investigates HRV and symbolic HR transition dynamics during sleep in individuals with T2DM, aiming to enhance understanding of how autonomic regulation during sleep relates to diabetic complications. Using frequency-domain HRV measures and symbolic motif analysis, distinct differences in cardiac autonomic patterns were identified between daytime and sleep. More importantly, HR transition motifs derived during sleep exhibited stronger associations with diabetic complications than those measured during the daytime. These findings underscore the importance of assessing autonomic function specifically during sleep and add to evidence emphasizing sleep as a sensitive window for detecting subclinical cardiovascular dysfunction in metabolic disorders.

In this study, we found that the HFp during sleep was significantly higher than during the daytime (Fig 2), indicating that parasympathetic tone is dominant during nocturnal sleep [24]. However, the LFp and VLFp did not show significant changes, suggesting that slower oscillatory HRV may be less sensitive to circadian influences or represent more stable autonomic regulation.

Beyond frequency-based measures, symbolic HR motif analysis provided additional insight into short-term HR transitions. Motifs such as [1, 1, −1] and [−1, 1, 1], which encode sequences of acceleration and deceleration in HR over three beats, showed significantly different prevalence during sleep compared with daytime (Fig 3), and exhibited strong positive correlations with HFp (Table 3), suggesting distinct physiological control mechanisms at night.

Motif prevalence showed the strongest correlations with diabetes-related complications during sleep, underscoring the unique value of sleep-specific HRV measures in metabolic health monitoring. Previous research has demonstrated that HRV patterns across different sleep stages are closely linked with metabolic function and glycemic control in T2DM [25]. For instance, Bhati et al. [26] reported that inflammatory and endothelial biomarkers were significantly associated with impaired cardiac vagal control and HRV in patients with type 2 diabetes, suggesting a pathophysiological link between subclinical inflammation, endothelial dysfunction, and autonomic impairment.

The physiological rationale for this phenomenon lies in the natural circadian rhythm of the ANS. During sleep, especially in stable non-rapid eye movement (REM) stages, environmental and behavioral confounders such as activity, posture, and emotional stress are minimized. This creates a more internally regulated state, predominantly governed by the parasympathetic system. Consequently, subtle disruptions in vagal function that may be masked or compensated for during the day become more apparent at night. In T2DM, early autonomic neuropathy typically involves vagal fibers, resulting in reduced HFp and elevated resting heart rate [8,27]. Our findings suggest that symbolic HR motifs are particularly sensitive to these early changes, providing a non-invasive marker of autonomic impairment.

Furthermore, sleep serves as an essential regulator of metabolic homeostasis. Sleep disruptions enhance insulin resistance and glucose intolerance, whereas inadequate glycemic management impairs sleep architecture [28,29]. In this context, we speculate that motif dynamics may act as an indicator of the reciprocal relationship between autonomic tone and metabolic regulation. The correlation between motifs and HFp, along with vagal activity, may indicate the body’s ability to adapt and recover from metabolic stressors during sleep.

Another important observation is the specificity of these correlations. Traditional HRV metrics, such as root mean square of successive differences (RMSSD) and the LF/HF ratio, provide broad estimates of autonomic tone, but lack the temporal resolution and physiological interpretability of symbolic dynamics [14,30,31]. In contrast, symbolic analysis converts continuous HR signals into discrete patterns, allowing detection of physiologically relevant rhythm transitions that frequency-domain methods may not capture [16].

To assess the robustness of our findings, we conducted several sensitivity analyses. When excluding participants with coronary artery disease or cardiac insufficiency (S1 Table), and when excluding those taking sleep medication (S2 Table), the sleep-based motif models remained statistically significant, particularly models with [1, 1, −1]. In addition, incorporating HbA1c as a covariate (S3 Table) did not alter the results.

The increased sensitivity of symbolic motifs to diabetic complications has clinical implications. Current diagnostic tools for diabetic autonomic neuropathy (DAN) rely on Ewing’s cardiovascular reflex tests, but are time-consuming and patient-dependent [32]. Our results suggest that sleep-derived HR motifs could serve as early biomarkers for subclinical DAN, potentially enabling earlier intervention. Furthermore, the non-invasive, low-cost nature of ECG monitoring makes this approach feasible for large-scale screening, especially when integrated with wearable technologies or home-based health monitoring systems.

It is important to note that not all motifs showed significant associations with autonomic features or complications, underscoring the need to focus on physiologically meaningful patterns. In this study, [1,1,-1] and [−1,1,1] emerged as particularly relevant due to their structured transitions, which may reflect adaptive oscillations in cardiac control. By contrast, motifs representing static or unidirectional changes (e.g., [0,0,0] or [1,1,1]) may capture random noise or monotonous rhythms less indicative of healthy regulatory complexity. Future research should investigate the physiological basis of different motif classes and their potential links to autonomic reflexes or pathophysiological states.

## Limitations

This study has several limitations. The limited sample size, consisting solely of males, restricts the generalizability of the findings to other populations. Sleep was analyzed using broad day–night epochs rather than specific stages, which may have masked stage-dependent autonomic differences. The cross-sectional design also prevents causal inference, leaving ambiguity as to whether changes in motif dynamics occur prior to or as a consequence of diabetic complications. Finally, the exclusion of certain clinical variables due to missing data may have further constrained the scope of the analysis.

Future research should address these limitations by recruiting larger and more diverse cohorts, incorporating detailed sleep staging and multi-night recordings, and adopting longitudinal designs to clarify temporal relationships. Extensions of this work could combine symbolic motif analysis with additional physiological signals, such as respiration or skin conductance, to better characterize the autonomic substrates underlying motif patterns. In addition, symbolic motif analysis can be extended to assess coupling or synchronization between two physiological signals, enabling the distinction between normal and abnormal patterns, as demonstrated in previous studies [33–35].

## Conclusion

This study demonstrates that symbolic HR transition motifs during sleep provide meaningful insights into autonomic regulation in individuals with T2DM. Compared to conventional HRV metrics, specific motifs, particularly [1,1,-1] and [−1,1,1], exhibited strong associations with parasympathetic activity and were predictive of diabetic complications. These findings underscore the importance of evaluating sleep-specific autonomic patterns and support the use of symbolic motif analysis as a non-invasive biomarker for subclinical autonomic dysfunction.

### Declaration of generative AI and AI-assisted technologies in the writing process

The authors utilized ChatGPT to assist with language editing and text refinement during the preparation of this manuscript. All content was subsequently reviewed and revised by the authors, who take full responsibility for the final version of the publication.

## Supporting information

S1 Table Comparison of daytime and sleep heart rate (HR) transitions and high-frequency (HF) components in relation to their correlation with diabetic complications (Excluding patients with coronary artery disease and cardiac insufficiency). n = 33 (patients with diabetic complications = 14). (DOCX)

S2 Table Comparison of daytime and sleep heart rate (HR) transitions and high-frequency (HF) components in relation to their correlation with diabetic complications (Excluding patients who are taking sleep medication). n = 27 (patients with diabetic complications = 11). (DOCX)

S3 Table Comparison of daytime and sleep heart rate (HR) transitions and high-frequency (HF) components in relation to their correlation with diabetic complications with including HbaA1c. n = 24 (patients with diabetic complications n = 13). (DOCX)

S1 File Sleep questionnaire. (XLSX)
