# Supplementary material for: Symbolic heart rate transition motifs during nocturnal sleep are associated with diabetic complications in type 2 diabetes
Source: PLoS One. 2025 Sep 24;20(9):e0333067. doi: 10.1371/journal.pone.0333067 (PMC12459800; doi:10.1371/journal.pone.0333067)
Supplement: S1 Table — (DOCX) [file pone.0333067.s001.docx]

**Supplementary Table 1:** Comparison of daytime and sleep heart rate (HR) transitions and high-frequency (HF) components in relation to their correlation with diabetic complications (Excluding patients with coronary artery disease and cardiac insufficiency). n = 33 (patients with diabetic complications = 14).

| **Feature** | **Day** | | | | **Sleep** | | | |
| --- | --- | --- | --- | --- | --- | --- | --- | --- |
|  | **β** | ***P* - value** | **95% CI** | **Model P-value** | **β** | ***P* - value** | **95% CI** | **Model *P*-value** |
| **Model 1** | | | | | | | | |
| Age | 0.07 | 0.890 | [-0.99, 1.1] | 0.140 | - 0.43 | 0.530 | [-1.8, 0.97] | 0.070 |
| BMI | - 0.85 | 0.110 | [-1.9, 0.24] |  | -0.95 | 0.080 | [-2.1, 0.17] |  |
| HFp | - 0.17 | 0.690 | [-1.1, 0.73] |  | - 0.79 | 0.220 | [-2.1, 0.53] |  |
| **Model 2** | | | | | | | | |
| Age | 0.20 | 0.700 | [-0.86, 1.3] | 0.150 | - 0.61 | 0.350 | [-2.0, 0.73] | 0.009 |
| BMI | - 0.83 | 0.120 | [-1.9, 0.26] |  | - 1.10 | 0.050 | [-2.2, 0.07] |  |
| [1, 1, -1] | 0.09 | 0.820 | [-0.75, 0.94] |  | -1.60 | 0.040 | [-3.2, 0.00] |  |
| **Model 3** | | | | | | | | |
| Age | 0.07 | 0.890 | [-1.01, 1.2] | 0.150 | - 0.44 | 0.480 | [-1.7, 0.82] | 0.030 |
| BMI | - 0.88 | 0.100 | [-2.0, 0.22] |  | - 1.00 | 0.070 | [-2.1, 0.11] |  |
| [-1, 1, 1] | - 0.14 | 0.740 | [-1.1, 0.72] |  | -1.10 | 0.100 | [-2.4, 0.26] |  |

BMI: body mass index, HFp: high frequency power, CI: Confidence Interval.
