# Supplementary material for: Symbolic heart rate transition motifs during nocturnal sleep are associated with diabetic complications in type 2 diabetes
Source: PLoS One. 2025 Sep 24;20(9):e0333067. doi: 10.1371/journal.pone.0333067 (PMC12459800; doi:10.1371/journal.pone.0333067)
Supplement: S2 Table — (DOCX) [file pone.0333067.s002.docx]

**Supplementary Table 2:** Comparison of daytime and sleep heart rate (HR) transitions and high-frequency (HF) components in relation to their correlation with diabetic complications (Excluding patients who are taking sleep medication). n = 27 (patients with diabetic complications = 11).

| **Feature** | **Day** | | | | **Sleep** | | | |
| --- | --- | --- | --- | --- | --- | --- | --- | --- |
|  | **β** | ***P* - value** | **95% CI** | **Model P-value** | **β** | ***P* - value** | **95% CI** | **Model *P*-value** |
| **Model 1** | | | | | | | | |
| Age | 0.76 | 0.180 | [-0.41, 1.9] | 0.100 | 0.28 | 0.720 | [-1.4, 1.9] | 0.070 |
| BMI | - 0.39 | 0.480 | [-1.5, 0.76] |  | - 0.54 | 0.350 | [-1.7, 0.66] |  |
| HFp | - 0.19 | 0.700 | [-1.2, 0.81] |  | - 0.67 | 0.350 | [-2.1, 0.80] |  |
| **Model 2** | | | | | | | | |
| Age | 1.00 | 0.110 | [-0.28, 2.3] | 0.090 | - 0.34 | 0.650 | [-2.0, 1.3] | 0.008 |
| BMI | - 0.33 | 0.560 | [-1.5, 0.85] |  | - 0.92 | 0.160 | [-2.3, 0.42] |  |
| [1, 1, -1] | 0.28 | 0.560 | [-0.72, 1.3] |  | -1.90 | 0.050 | [-3.3, 0.12] |  |
| **Model 3** | | | | | | | | |
| Age | 0.78 | 0.190 | [-0.44, 2.0] | 0.110 | 0.09 | 0.900 | [-1.5, 1.7] | 0.040 |
| BMI | - 0.40 | 0.470 | [-1.6, 0.76] |  | - 0.68 | 0.250 | [-1.9, 0.56] |  |
| [-1, 1, 1] | - 1.10 | 0.820 | [-1.1, 0.89] |  | -1.01 | 0.180 | [-2.6, 0.58] |  |

BMI: body mass index, HFp: high frequency power, CI: Confidence Interval.
