# Supplementary material for: Symbolic heart rate transition motifs during nocturnal sleep are associated with diabetic complications in type 2 diabetes
Source: PLoS One. 2025 Sep 24;20(9):e0333067. doi: 10.1371/journal.pone.0333067 (PMC12459800; doi:10.1371/journal.pone.0333067)
Supplement: S3 Table — (DOCX) [file pone.0333067.s003.docx]

**Supplementary Table 3:** Comparison of daytime and sleep heart rate (HR) transitions and high-frequency (HF) components in relation to their correlation with diabetic complications with including HbaA1c. n = 24 (patients with diabetic complications n = 13).

| **Feature** | **Day** | | | | **Sleep** | | | |
| --- | --- | --- | --- | --- | --- | --- | --- | --- |
|  | **β** | ***P* - value** | **95% CI** | **Model *P*-value** | **β** | ***P* - value** | **95% CI** | **Model *P*-value** |
| **Model 1** | | | | | | | | |
| Age | 0.66 | 0.350 | [-0.83, 2.2] | 0.660 | 0.16 | 0.850 | [-1.6, 1.9] | 0.120 |
| BMI | - 0.55 | 0.360 | [-1.8, 0.71] |  | -0.72 | 0.250 | [-2.0, 0.60] |  |
| HFp | - 0.11 | 0.820 | [-1.2, 0.92] |  | - 1.50 | 0.070 | [-3.2, 0.21] |  |
| Hb1Ac | 0.35 | 0.340 | [-0.42, 1.1] |  | 0.45 | 0.280 | [-0.45, 1.4] |  |
| **Model 2** | | | | | | | | |
| Age | 0.80 | 0.260 | [-0.68, 2.3] | 0.640 | 0.07 | 0.940 | [-13, 4.6] | 0.035 |
| BMI | - 0.47 | 0.450 | [-1.8, 0.83] |  | - 0.83 | 0.210 | [-2.2, 0.55] |  |
| [1, 1, -1] | 0.18 | 0.720 | [-0.9, 1.3] |  | -1.90 | 0.030 | [-3.8, -0.11] |  |
| Hb1Ac | 0.28 | 0.450 | [-0.50, 1.1] |  | 0.50 | 0.300 | [-0.51, 1.5] |  |
| **Model 3** | | | | | | | | |
| Age | 0.60 | 0.400 | [-0.87, 2.1] | 0.590 | -0.06 | 0.940 | [-1.9, 1.7] | 0.220 |
| BMI | - 0.53 | 0.380 | [-1.8, 0.71] |  | - 0.71 | 0.260 | [-2.0, 0.60] |  |
| [-1, 1, 1] | - 0.30 | 0.510 | [-1.3, 0.66] |  | -1.30 | 0.100 | [-2.9, 0.37] |  |
| Hb1Ac | 0.36 | 0.310 | [-0.38, 1.1] |  | 0.38 | 0.320 | [-0.43, 1.2] |  |

BMI: body mass index, HFp: high frequency power, CI: Confidence Interval.
